# Sustained high body temperature exacerbates cognitive function and Alzheimer’s disease-related pathologies

**DOI:** 10.1038/s41598-022-16626-0

**Published:** 2022-07-18

**Authors:** Cha-Gyun Jung, Reiko Kato, Chunyu Zhou, Mona Abdelhamid, Esraa Ibrahim A. Shaaban, Hitoshi Yamashita, Makoto Michikawa

**Affiliations:** 1 Department of Biochemistry, Graduate School of Medical Sciences, Nagoya City University, 1 Kawasumi, Mizuho-cho, Mizuho-ku, Nagoya, 467-8601 Japan; 2 Department of Biomedical Sciences, College of Life and Health Sciences, Chubu University, 1200 Matsumoto-cho, Kasugai, 487-8501 Japan

**Keywords:** Alzheimer's disease, Cognitive ageing

## Abstract

Global warming is a serious public health threat to people worldwide. High body temperature is one of the important risk factors for Alzheimer’s disease (AD), and the body temperature of AD patients has been found to be significantly higher than that of elderly control subjects. However, the effects of high body temperature on cognitive function and AD pathologies have not been completely elucidated. We report here that Tg2576 mice housed at a high ambient temperature of 30 °C for 13 months showed an increase in the body temperature, which is accompanied by memory impairment and an enhancement of amyloid-β peptides (Aβ) generation through the upregulation of β-site APP cleaving enzyme 1 (BACE1) level and decrease in the level of an Aβ-degrading enzyme, neprilysin (NEP) in the brain, compared with those of Tg2576 mice at 23 °C. High body temperature also increased the levels of heat shock proteins (HSPs), stress-stimulated kinases such as JNK, and total tau, leading to the enhancement of tau phosphorylation at 30 °C. Taken together, our findings suggest that high body temperature exacerbates cognitive function and AD pathologies, which provides a mechanistic insight for its prevention.

## Introduction

Alzheimer’s disease (AD) is characterized by two prominent histopathological features, namely, extracellular amyloid-β peptides (Aβ) deposition^1^ and presence of intraneuronal neurofibrillary tangles of aberrant hyperphosphorylated tau protein in neurons^2^, which lead to synaptic loss, neuronal loss, and memory impairment. Sporadic late-onset AD (SAD) is considered to be the major type of AD, which may result from an imbalance between Aβ generation and Aβ degradation/clearance in the brain^3^. Although the cause of SAD remains unclear, it has been commonly accepted that external or environmental factors are associated with SAD and accelerate the development of the disease^4^. Among risk factors for AD, aging is the greatest risk factor, leading to marked increases in the incidence of AD among the elderly. AD patients also show some symptoms of noncognitive disorders such as hyperactivity, anxiety, depression, agitation, and disturbed sleep and circadian rhythms, which adversely affect the quality of life^5–8^. Moreover, global warming is one of the serious public health threats to people worldwide. A sustained rise in environmental temperature largely affects body temperature regulation and causes an increase in body temperature. However, it is unknown whether a sustained high body temperature itself affects AD pathologies, irrespective of sleep disturbances.

It has been suggested that a low body temperature is one of the central risk factors for AD, and several reports indicated that elderly people showed a marked decrease in body temperature^9^. This is likely a consequence of impaired thermogenesis and thermoregulation due to an age-related decline in respiratory, neuromuscular, and gastrointestinal functions^10^. Although molecular mechanisms underlying the association of a low body temperature with AD pathogenesis have not been completely clarified, animal studies have shown that a low body temperature induces the phosphorylation of tau^11^ and that anesthesia reduces the body temperature leading to an increase in the levels of phosphorylated (p)-tau^12^. This study also showed that acute hypothermia induced by anesthesia has no effect on APP metabolism including no changes in Aβ level during anesthesia, suggesting that hypothermia might not be involved in Aβ generation. On the other hand, it has also been reported that the body temperature in patients with AD was significantly higher than that in healthy elderly subjects, and a higher body temperature might have an adverse impact on the disease. Although the consequences of a high body temperature on AD are not well elucidated, it has been suggested that AD affects hypothalamic function, which interferes with the regulation of body temperature, which in turn leads to disturbances of the regulation of body temperature and circadian rhythms of locomotor activities^13–15^. There is accumulating evidence that sleep disturbance is one of the markers for AD associated with cognitive impairment, and sleep deprivation alters the regulation of body temperature^6,8,14,16^. The body temperature is higher in the wake phase than in sleep, and sleep deprivation causes an increase in soluble Aβ level and enhancement of Aβ deposition in an animal model^16,17^. In addition, some in vitro studies suggested that an elevated temperature might have a negative effect on the disease as higher temperatures increase the expression of amyloid precursor protein (APP) and the rate of Aβ oligomerization and fibril formation^18–20^.

Recently, we have found that high temperature is associated with the formation of the γ-complex, which enhances Aβ generation in culture^21^. However, the effects of high body temperature on cognitive function and AD-like pathologies have not well analyzed. Therefore, we investigated the effects of high body temperature on cognitive function and AD pathologies, such as those associated with Aβ plaques and tau phosphorylation, using Tg2576 mice, an AD-like mouse model.

## Results

### Effect of high ambient temperature on core body temperature and physical activity

**Figure 1:**
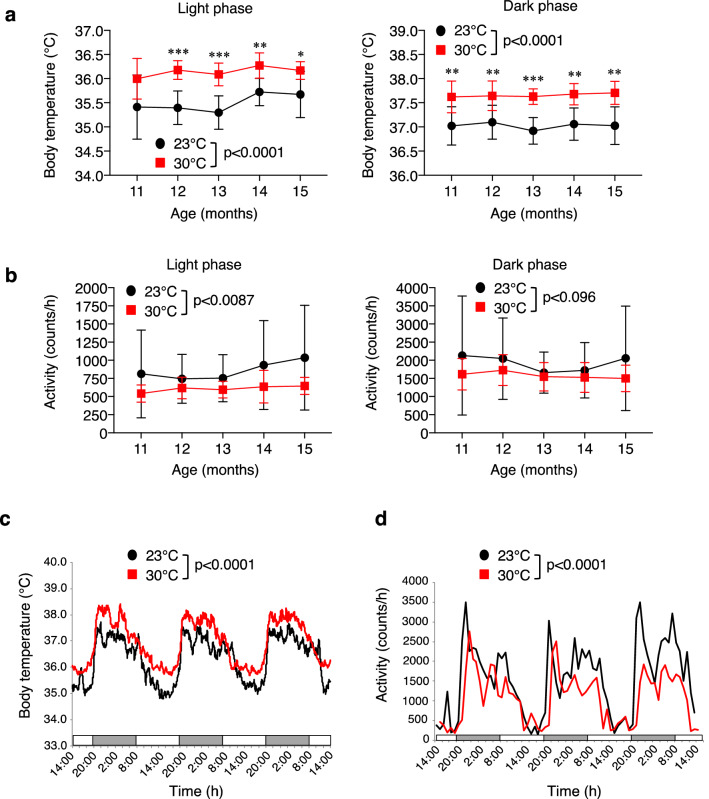
High-ambient-temperature exposure increases core body temperature. (**a**, **b**) Core body temperature and physical activity of Tg2576 mice housed at 23 °C or 30 °C were analyzed every month from 11 to 15 months using a VitalView system. Average core body temperature (**a**) and physical activity (**b**) of mice in light and dark phases at ambient temperatures of 23 °C and 30 °C (*n* = 7 for each group). (**c**, **d**) Circadian rhythm of average core body temperature (**c**) and physical activity (**d**) at 15 months of age (*n* = 7 for each group). Data are expressed as mean ± SD. Significant differences among groups were assessed by repeated measures ANOVA. *p < 0.05, **p < 0.01, ***p < 0.001 vs 23 °C.

It is well unknown whether high ambient temperature can affect cognitive function and AD pathologies, as well as Aβ levels and tau phosphorylation in the brain. Therefore, we studied the effects of high ambient temperature on cognitive function and AD-like pathologies under the higher ambient temperature of 30 °C using Tg2576 mic. We first measured the core body temperature of mice using a non-invasive monitoring system at 11, 12, 13, 14, and 15 months of age. Both mouse groups housed either at 23 °C or 30 °C exhibited circadian rhythms in core body temperature with the temperature being highest during the dark phase at all ages examined. Compared with the normal room temperature of 23 °C, however, the core body temperature was significantly higher (0.50–0.79 °C) during the dark and light phases in the mice housed at 30 °C at all ages examined (Fig. 1a and c). Conversely, as expected, physical activity from 11 to 15 months decreased in the light phase, but not in the dark phase, at 30 °C than at 23 °C (Fig. 1b) and circadian rhythms of physical activity was significantly lower at 30 °C than at 23 °C at 15 months of age (Fig. 1d). These findings indicate that the long-term exposure of Tg2576 mice to high ambient temperature (30 °C) resulted in a sustained high body temperature with a decrease in physical activity.

### Effect of high ambient temperature on cognitive function

**Figure 2:**
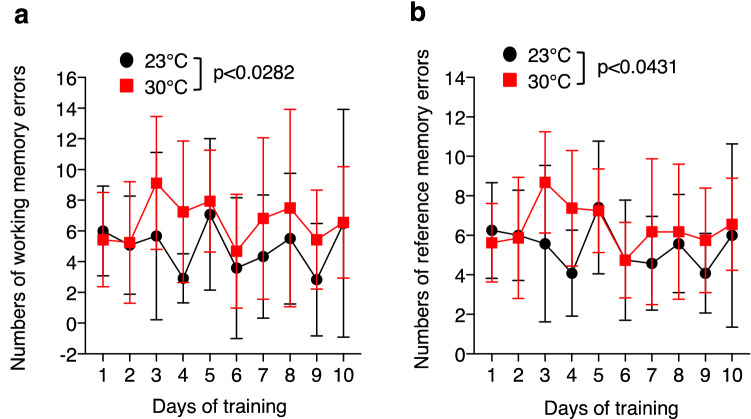
High-ambient-temperature exposure induces spatial memory impairment. Memory of Tg2576 mice housed at 23 °C or 30 °C were analyzed using the radial arm maze task at 16 months of age. Mean numbers of working memory errors (**a**) and reference memory errors (**b**) in the radial arm maze task (*n* = 6 for 23 °C, *n* = 8 for 30 °C). Data are expressed as mean ± SD. Significant differences among groups were assessed by repeated measures ANOVA.

Next, we determined whether high ambient temperature could affect cognitive function. To verify the impact of the high ambient temperature of 30 °C on the spatial/working memory of Tg2576 mice at 17 months of age, we subjected the mice to the radial arm maze task, which was previously used to characterize memory impairment in Tg2576 mice^22^. We firstly evaluated the working memory errors of the two groups. Initially, the memory acquisition ability of all the mice was poor and the number of working memory errors occurring was larger in the mice housed at 30 °C than in those housed at 23 °C (Fig. 2a). We further assessed cognitive function on the basis of reference memory errors. Compared with the ambient temperature at 23 °C, the number of reference memory errors occurring was larger in the mice housed at 30 °C (Fig. 2b). Taken together, these results indicate that the high ambient temperature induces memory impairment.

### Effect of high ambient temperature on Aβ deposition and generation

**Figure 3:**
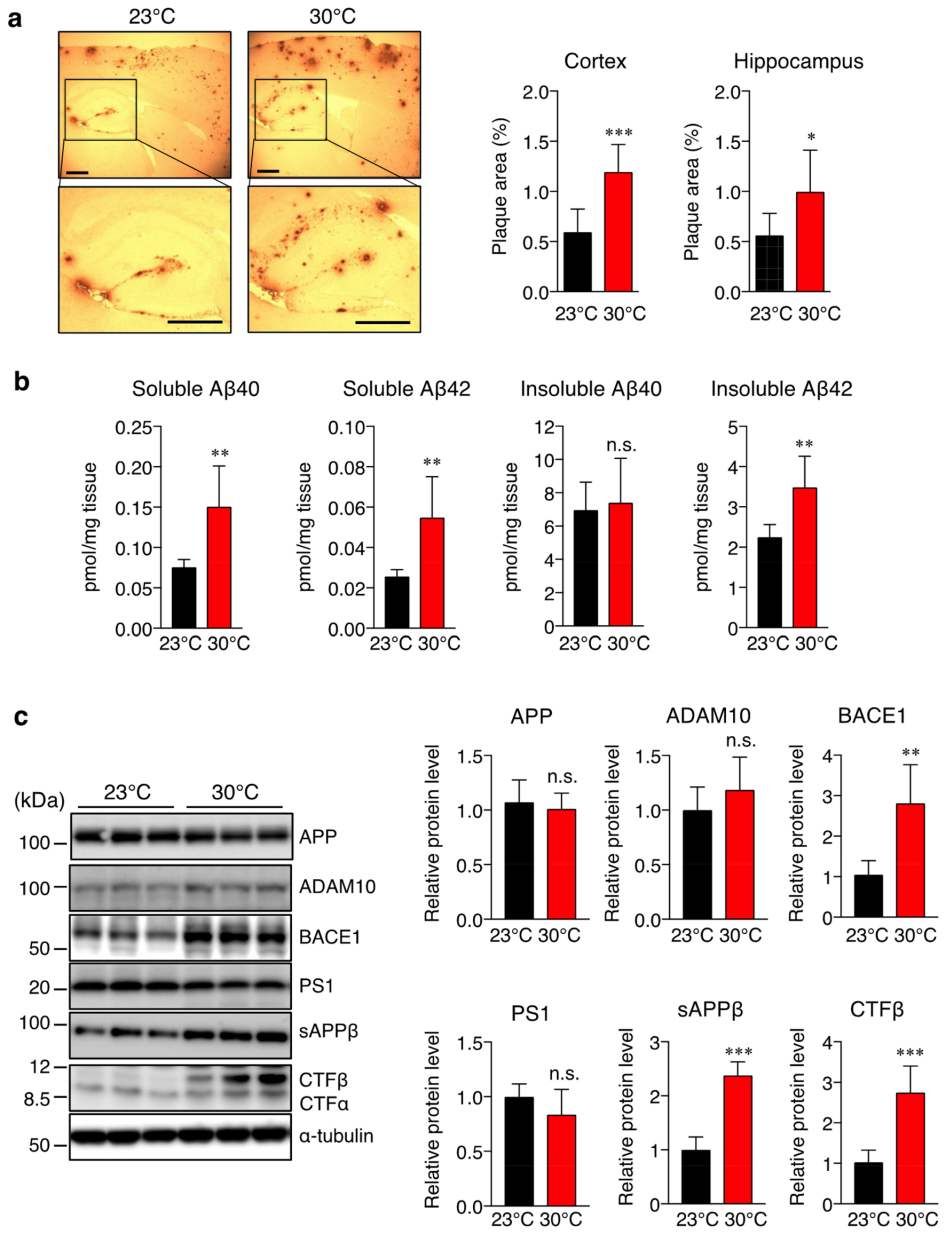
High-ambient-temperature exposure increases Aβ deposition and Aβ levels in the brain cortex via the upregulation of BACE1. After the physiological analyses, brain samples of 17-month-old Tg2576 mice housed at 23 °C or 30 °C were used for Aβ staining, sandwich ELISA, and Western immunoblotting. (**a**) Sagittal brain sections of mice were stained with the anti-Aβ antibody (82E1) recognizing both Aβ40 and Aβ42 to detect Aβ deposition. Representative images are shown on the left panel. Aβ deposits in the cortex and hippocampus were quantified as the percentage of the immunostained area with respect to the total area examined (right panel). (**b**) Soluble and insoluble Aβ40 and Aβ42 levels in the brain cortex were measured by sandwich ELISA. Aβ levels were normalized to brain tissue weight. (**c**) Protein levels of APP, ADAM10, BACE1, PS1, sAPPβ, CTFβ, and α-tubulin in the brain cortex were determined by Western immunoblotting. Representative immunoblots and relative protein levels quantified by densitometry are shown in the left and right panels, respectively. The data are expressed as mean ± SD, *n* = 6 for 23 °C, *n* = 6 for 30 °C, *p < 0.05, **p < 0.01, ***p < 0.001 vs 23 °C, n.s., no significant difference, as determined by Student’s *t*-test. Scale bars: 500 μm.

Next, we further examined whether the memory impairment at a high ambient temperature of 30 °C correlates with its effect on amyloid pathology in the brain of Tg2576 mice. We assessed the total Aβ plaques in both cortical and hippocampal tissues of the mice at 17 months of age after the radial arm maze task. The anti-82E1 antibody can recognize both Aβ40 and Aβ42, so brain sections were stained with this antibody. The mice housed at 30 °C showed a significantly higher level of Aβ deposition in both the cortex and hippocampus than the mice housed at 23 °C (Fig. 3a). Quantitative analysis of the signal intensities demonstrated that the levels of Aβ deposition in the cortex and hippocampus increased by ~ 187% and ~ 222%, respectively, at 30 °C compared with those at 23 °C (Fig. 3a). To validate these observations on Aβ deposition, we further carried out Aβ ELISA to measure the levels of soluble and insoluble Aβ40 and Aβ42 in the cortical homogenates of mice. Results revealed that the mice housed at 30 °C showed higher levels of cortical soluble Aβ40 and Aβ42 than those at 23 °C (Fig. 3b), which suggests that a high ambient temperature promotes Aβ deposition and generation. To investigate the mechanisms underlying this temperature-mediated increase in Aβ generation, we measured APP and APP-cleaving enzyme levels in the cortical homogenates of the mice by Western blotting. We found that the BACE1 (β-secretase) level was significantly higher at 30 °C than that at 23 °C (Fig. 3c). However, there were no significant differences in the levels of APP, ADAM10 (α-secretase), and PS1 (γ-secretase component) between the two groups (Fig. 3c). We further measured the levels of soluble APPβ (sAPPβ) and APP cleavage fragments, including the C-terminal fragment α (CTFα) and CTFβ of APP in the cortex of mice. As expected, both sAPPβ and CTFβ levels were significantly higher at 30 °C than at 23 °C (Fig. 3c). Interestingly, although the insoluble Aβ40 level in the cortex was not significantly different between the two groups, the insoluble Aβ42 level was significantly higher at 30 °C than at 23 °C (Fig. 3b). Taken together, these findings suggest that the amyloidogenic processing of APP is enhanced through the increase in the BACE1 level in the brain of Tg2576 mice with a sustained high body temperature.

### Effect of high ambient temperature on Aβ degradation and clearance

**Figure 4:**
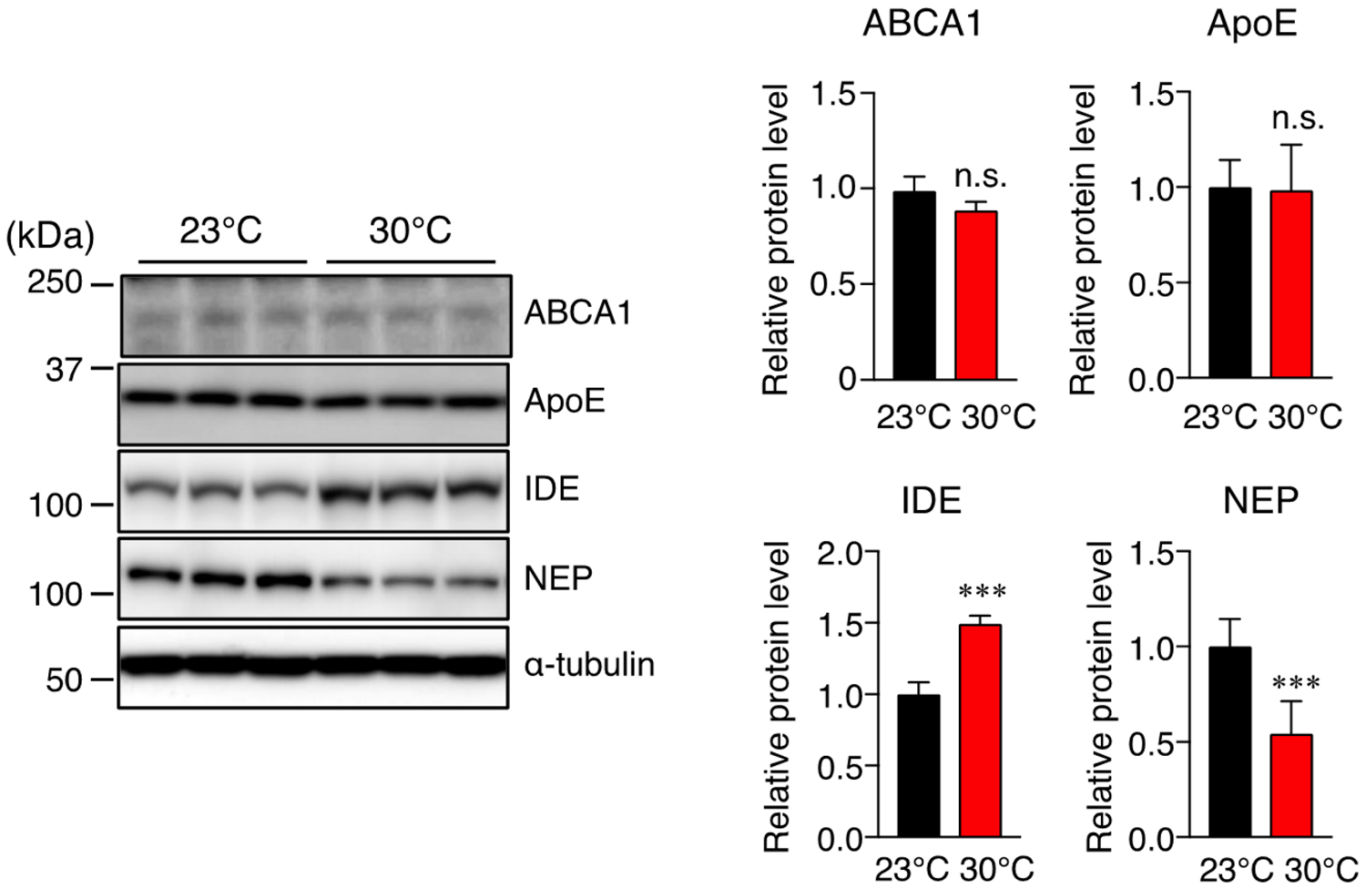
High-ambient-temperature exposure increases IDE level and decreases NEP level in the brain cortex. Protein levels of ABCA1, ApoE, IDE, NEP and α-tubulin in the brain cortex of 17-month-old mice housed at 23 °C or 30 °C were determined by Western immunoblotting. Representative immunoblots and relative protein levels quantified by densitometry are shown in the left and right panels, respectively. The data are expressed as mean ± SD, *n* = 6 for 23 °C, *n* = 6 for 30 °C, **p < 0.01, ***p < 0.001 vs 23 °C, n.s., no significant difference, as determined by Student’s *t*-test.

As we have shown above, although the level of insoluble Aβ40 in the cortex was not significantly different between the two groups, the level of insoluble Aβ42 was significantly higher at 30 °C than at 23 °C, which indicates that the sustained high body temperature may contribute to the pathogenic accumulation of Aβ42 in the cortex. It has been well documented that on the basis of the amyloid hypothesis, the upregulation of Aβ accumulation is mainly caused by two mechanisms: Aβ overproduction or the reduction of Aβ proteolytic degradation and impaired Aβ clearance^3^. Thus, we measured the levels of an insulin-degrading enzyme (IDE) and neprilysin (NEP), which are Aβ-degrading enzymes. We also measured the levels of ATP-binding cassette A1 (ABCA1) and apolipoprotein E (ApoE), which are Aβ clearance-related proteins, in the cortex of mice by Western blotting. Interestingly, we found that although the IDE level significantly increased, the NEP level significantly decreased at 30 °C compared with those at 23 °C (Fig. 4). We observed no significant differences in the levels of ApoE and ABCA1 between the two groups (Fig. 4). These findings indicate that the decreased cortical NEP levels in mice housed at 30 °C may be attributable to Aβ42 accumulation.

### Effect of high ambient temperature on tau pathology

**Figure 5:**
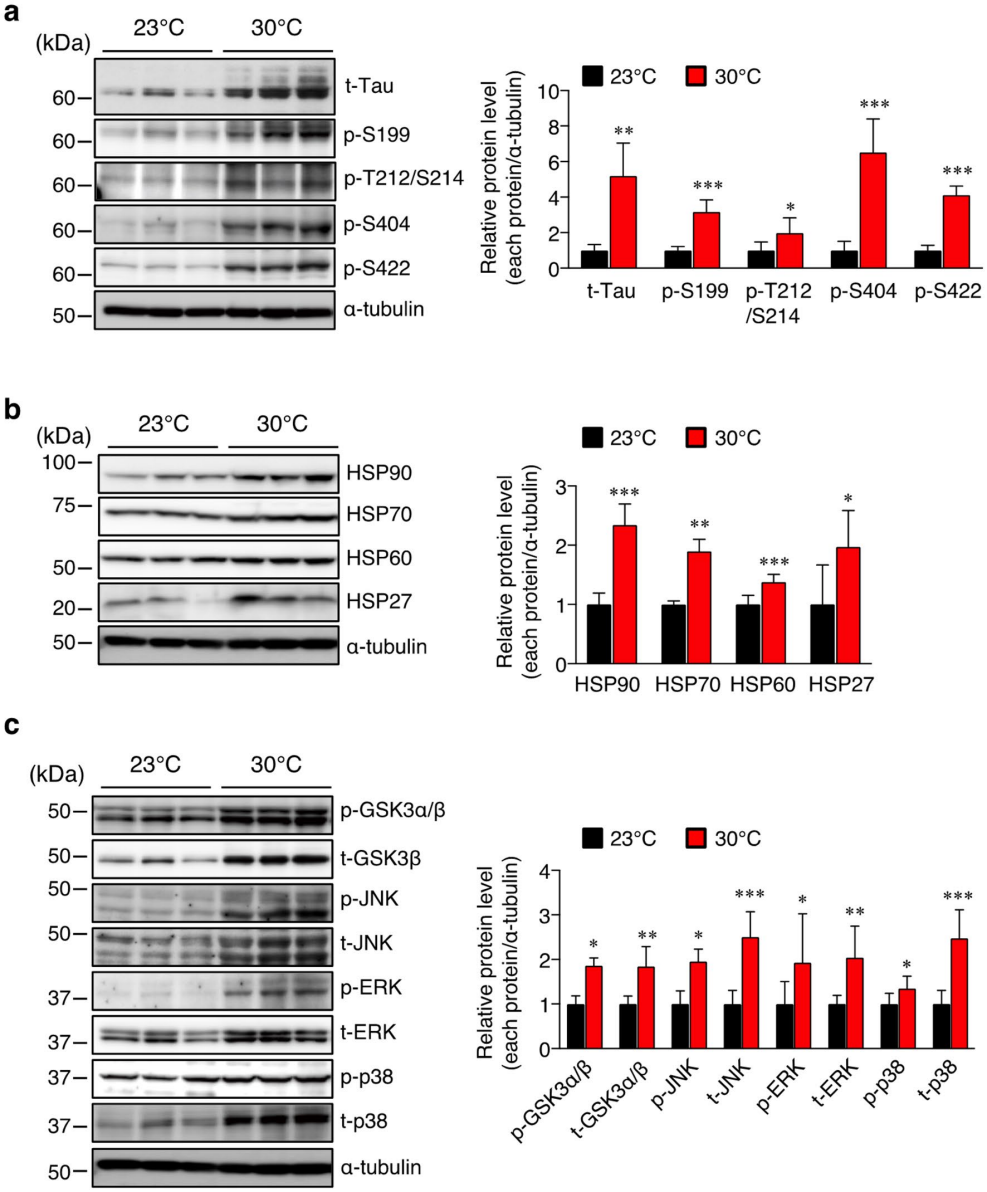
High-ambient-temperature exposure increases the levels of total tau, heat shock proteins, and total protein kinases in the cortex. Western immunoblotting was performed using the brain cortex of 17-month-old mice. (**a**) Protein levels of total- (t-) and phosphorylated- (p-) tau on multiple sites (Ser199, Thr212/Ser214, Ser404, and Ser422), and α-tubulin. (**b**) Protein levels of HSP90, HSP70, HSP60, and HSP27, and α-tubulin. (**c**) Protein levels of total (t-) and phosphorylated-(p-) GSK3β, JNK, ERK, and p38. Representative immunoblots and relative protein levels quantified by densitometry are shown in the left and right panels, respectively. The data are expressed as mean ± SD, *n* = 6 for 23 °C, *n* = 7 for 30 °C, *p < 0.05, **p < 0.01, ***p < 0.001 vs 23 °C, n.s., no significant difference, as determined by Student’s *t*-test.

The pathological hyperphosphorylation and aggregation of tau are causal factors that induce synaptic loss and neurodegeneration, leading to memory impairment in patients with AD. It has been reported that excessive tau phosphorylation at Ser199, Thr212, Ser214, Ser404, and Ser422 inhibits physiological tau binding to microtubules, resulting in the impairment of their physiological function^23^. Therefore, we examined whether a high ambient temperature could affect tau phosphorylation. We determined the levels of total and phosphorylated (p)-tau on multiple sites (Ser422, Ser404, Thr212/Ser214, and Ser199) in the cortex of mice by Western blotting. Interestingly, the total tau level was significantly higher at 30 °C than that at 23 °C, which is accompanied by an enhancement of tau phosphorylation at pS199, T212/S214, pS404, and pS422 epitopes (Fig. 5a). These findings suggest that a sustained high body temperature may increase tau stability, which consequently leads to an increase in p-tau level. We then investigated the molecular mechanisms underlying the increase in total tau level in the cortex of Tg2576 mice. Many studies have provided evidence that two major molecular chaperones, heat shock protein (HSP) 70 and HSP90, are involved in tau stability by affecting tau misfolding, self-aggregation, and clearance^24–27^. As expected, the levels of all the HSPs (HSP90, HSP70, HSP60, and HSP27) examined were higher in the cortex of mice housed at 30 °C than in those housed at 23 °C (Fig. 5b). These findings indicate that the increase in the levels of these HSPs induced by the high ambient temperature may stabilize the tau protein. In tissues including the brain, heat shock stress responses also induce the activation of GSK3α/β and mitogen-activated protein kinase (MAPK) family members, such as the extracellular signal-regulated kinase (ERK), c-jun N-terminal kinase (JNK), and p38^28,29^. These kinases are also considered to be major physiological and pathological tau kinases. Therefore, we examined the expression and phosphorylation of these four kinases in the cortex of mice by Western blotting. Similarly, to total tau levels, the total levels of GSK3β, JNK, ERK, and p38 were significantly higher at 30 °C than at 23 °C (Fig. 5c). As the levels of these kinases increased, the phosphorylation levels of GSK3β, ERK (Thr202/Tyr204), JNK (Thr183/Tyr185), and p38 (Thr180/Tyr182) were also higher at 30 °C than at 23 °C (Fig. 5c). These results suggest that sustained increase in body temperature stimulates induction of HSPs, which resulted in the stabilization of proteins including tau and protein kinases, leading to tau hyperphosphorylation in the cortex of Tg2576 mice.

## Discussion

Accumulating evidence suggests that patients with AD often exhibit a higher core body temperature than healthy elderly control subjects, and prolonged or excessive hyperthermia can damage CNS^13–15^. Furthermore, sleep deprivation seen in AD is associated with a raised body temperature, which may cause cognitive impairment. However, the molecular mechanism(s) by which a high body temperature contributes to AD pathologies, such as those associated with Aβ plaques and tau phosphorylation, remain unknown. Here, we found that the long-term exposure of Tg2756 mice to the thermal condition of 30 °C led to a sustained high body temperature (about 0.50–0.79 °C) without an increase in locomotor activity associated with sleep deprivation. This small but sustained high body temperature in our models appears to be distinct from hyperthermia caused by failure of thermoregulation. However, this sustained high body temperature induced memory impairment through the increase in Aβ generation, deficits of Aβ42 degradation, the increase in HSPs, the induction of tau stabilization resulting in the hyperphosphorylation of tau, and kinase phosphorylation related to heat stress.

Although little is known about the causal relationship between high body temperature and cognitive dysfunction, animal and human studies have indicated that an elevated core body temperature has a negative effect on AD through increases in Aβ levels and enhancement of Aβ deposition in the brain, leading to memory impairment^18–20^. Regarding Aβ pathologies, we found that a sustained high body temperature increased the soluble Aβ40 and Aβ42 levels through an increase in BACE1 level. Aβ is generated by the proteolytic cleavage of APP by β- and γ-secretases, and it can cause synaptic and neuronal losses, which causes the impairment of cognitive function including spatial memory^1^. Furthermore, the increases in BACE1 expression level and activity are found in the brain of AD patients^30^. Oxidative stress is associated with an early onset of AD pathogenesis and it increases the expression level and enzyme activity of BACE1^31^. Although we did not obtain direct evidence of the induction of oxidative stress in the brain of Tg2576 mice housed at 30 °C, we found that the sustained high body temperature increased the total levels of stress-activated protein kinases, such as JNK, p38, and ERK kinases, and consequently led to the enhancement of their phosphorylation. Several studies have demonstrated that the activation of JNK may be linked to the induction of BACE1^32,33^. The activation of JNK is known to be an important factor in the pathogenesis of AD, which contributes to the enhancement of Aβ generation^32^ and tau phosphorylation^34^. Therefore, an increased BACE1 level induced by the sustained high body temperature may be, in part, a consequence of oxidative-stress-induced activation of JNK, leading to the enhancement of Aβ generation.

We also observed that although both soluble Aβ40 and Aβ42 levels were increased by the sustained high body temperature-induced BACE1 level, the levels of insoluble Aβ40 and Aβ42 were unchanged and increased, respectively. These results suggest that a sustained high body temperature is involved in Aβ degradation and/or clearance. It has been suggested that AD pathologies are linked to an imbalance between Aβ generation and removal including Aβ degradation and clearance. Among several enzymes involved in the degradation and clearance of Aβ in the brain, IDE and NEP are major endogenous Aβ-degrading enzymes, and their levels and activities negatively correlate with Aβ accumulation^35,36^. In this study, we found that the sustained high body temperature increased IDE levels and decreased NEP levels. Although the mechanism is as yet unknown, one possibility is that the increase in soluble Aβ40 and Aβ42 levels caused by the sustained high body temperature may increase IDE levels, which may be a compensatory response to reduce Aβ levels in the brain^37^. Therefore, the increased IDE level by the sustained high body temperature could reduce both Aβ40 and Aβ42 levels. On the contrary, the reduction in NEP level could inhibit Aβ42 degradation because NEP degrades Aβ42 rather than Aβ40^38^. NEP is a predominant Aβ protease and its activity and expression level are decreased in AD, which in turn, inhibits Aβ42 degradation and subsequently results in the accumulation of Aβ42 in the brain^39^. However, it is unclear why the increased IDE level and the decreased NEP level increased only insoluble Aβ42. Mechanisms by which sustained high body temperature alters expression levels of IDE and NEP leading to elevation of insoluble Aβ42 will be addressed in the future.

Tau pathology, a principal hallmark of AD, is characterized by the presence of hyperphosphorylated tau in the brain. Post-translational modification and abnormal accumulation of tau promote tauopathy. The increase in hyperphosphorylated tau level observed in the brain of AD patients may result from a dysregulation of the activity of tau kinase or from a failure of their degradation. Some studies also suggested that aberrant tau aggregation in the brain of AD patients may result from impaired ubiquitination and degradation of tau mediated by chaperone proteins^40,41^. Molecular chaperone proteins, including HSPs, upregulated in the AD brain^42^, can modulate tau proteostasis, and the expression levels of these proteins are increased under stress conditions^43^. Moreover, some studies provided pieces of evidence that tau stability is closely dependent on two major molecular chaperones, HSP90 and HSP70, which are involved in tau misfolding, self-aggregation, and clearance^24–26,44^. Although HSP90 contributes to refolding or degradation of various aberrant misfolded proteins, it is also involved in the preservation of aberrant tau. In line with these observations, it has been reported that HSP90 and HSP70 inhibitors degrade abnormal tau and attenuate tauopathy^45,46^. These findings strongly suggest that the increased HSPs levels are closely associated with tau stabilization. In this study, we found that the sustained high body temperature increased the total tau level leading to an increased p-tau level, indicating that a sustained high body temperature may contribute to tau protein stabilization, and this tau stabilization may in part be due to increased HSP levels. Tau phosphorylation is also regulated by various protein kinases, including GSK3β, ERK, p38, and JNK, which are considered to be major physiological and pathological tau kinases^34^. Similar to tau, the protein level and activity of these kinases were increased in the cortex of Tg2576 mice with higher body temperature. Therefore, it is plausible that sustained increase in body temperature induced the expression of HSPs, which increased stability of tau and stress-activated kinases, leading to accumulation of p-tau in the brain of Tg2576 mice at 30 °C.

**Figure 6:**
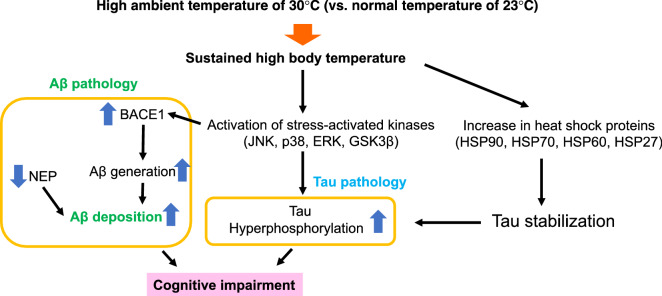
Proposed mechanisms of a sustained high body temperature-induced cognitive impairment. Sustained increase in body temperature increases BACE1 level through JNK activation and decreases NEP level, which exacerbates Aβ pathology. In addition, the sustained increase in body temperature activates stress-activated kinases and increases heat shock proteins leading to tau stabilization, which contribute to tau hyperphosphorylation.

In summary, this study demonstrates that a long-term exposure of Tg2576 mice to a high ambient temperature of 30 °C increases body temperature and memory impairment, which is associated with the enhancement of Aβ generation and deposition and tau hyperphosphorylation in the brain compared with those housed at 23 °C (Fig. 6). Our study highlights an impact of high body temperature on the progression of AD pathologies, providing a mechanistic insight for its prevention.

## Methods

### Experimental animals

Female Tg2576 mice overexpressing human APP695 with the Swedish mutation K670N/M671L were purchased from TACONIC (Hudson, NY). These mice have significantly high levels of insoluble brain Aβ at 6 months of age, which aggregates into Aβ plaques around 7 − 9 months of age^47^, and they show cognitive deterioration at 6 months of age^48^. Four-month-old Tg2576 mice were housed individually at 23 °C or 30 °C for 13 months in a 12-h light and dark cycle. All mice had free access to water and food, which was a standard chow (11.6% kcal from fat; Diet No. CE-2, CLEA Japan, Inc.), in our animal facility at 23 °C or 30 °C.

### Biotelemetry

A VitalView data acquisition system (Mini Mitter Co., Sunriver, Oregon, USA) was used to continuously record physical activity and core body temperature in unrestrained mice. In brief, we implanted intra-abdominally a temperature-sensitive transmitter (G2 E-Mitter, Mini Mitter Co.) after mice were anesthetized. Mice were allowed to recover for more than a week after implantation. VitalView software received the signals released by the transmitter (ER-4000, Mini Mitter Co.) and converted them into activity and temperature. Physical activity and core body temperature were monitored every minute and at a rate of 1 − 10 measurements per hour for 72 h, respectively.

### Radial arm maze task

The memory of the mice was examined using the eight-arm radial maze task as reported previously^22^. Briefly, the radial arm maze consisted of eight horizontal arms (31 × 9 cm) placed radially around a central platform (23 cm in diameter) above the floor. Mice were maintained in an 80–85% diet of ad libitum feeding for 7 days to restrict food before the task. Each mouse was exposed to working and reference memory tasks for 10 days after being placed individually in the center of the maze. The same four arms were baited for each daily training trial, while the other four arms were not used. The trials continued for 5 min or until the baits in the food cups in the four arms had been consumed. The behavior of mice in the trials was video-recorded and analyzed using a SMART Video Tracking System (Panlab s.l.u., Barcelona, Spain). The numbers of errors in the working memory task (entering an arm containing food that the mice had already entered before) and the reference memory task (entering an arm without a bait) were measured for each trial.

### Aβ ELISA

Inhalation of CO_2_ was used to kill the mice, which were then perfused with 0.1 M PBS. The brains of all mouse samples were removed and the cortical tissues of the left hemisphere were snap-frozen in liquid nitrogen and stored at − 80 °C until biochemical analysis, as described previously^49^. The cortical tissues were homogenized in 19 volumes of Tris-buffered saline (TBS; pH 7.6, 10 mM Tris and 150 mM NaCl) containing a phosphatase inhibitor cocktail (Wako Pure Chemical Industries, Osaka, Japan) and a protease inhibitor cocktail (Roche, Mannheim, Germany) and centrifuged at 100,000 rpm for 20 min at 4 °C. The supernatants were used for soluble Aβ measurements. After washing the pellets with TBS, the pellets were dissolved in 10 volumes of 6 M guanidine chloride, sonicated, and incubated for 1 h at RT. They were then centrifuged at 100,000 rpm for 20 min at 4 °C, and the resulting supernatants were used for insoluble Aβ measurements. Aβ40 and Aβ42 levels were assessed using ELISA kits (Wako) in accordance with the manufacturer’s instruction. The Aβ levels were normalized to the weight of the tissue.

### Immunohistochemical analysis

The right hemisphere of the brain was fixed in 4% buffered paraformaldehyde (PFA) solution, embedded in paraffin, and sectioned at 10 μm thickness. After the brain sections were deparaffinized, they were placed on slides and boiled in a pressure cooker containing 10 mM citrated buffer (pH 6.0) for antigen retrieval. Brain sections were incubated with an anti-Aβ (82E10, IBL, Gunma, Japan) antibody at 4 °C overnight after blocking with 5% normal goat serum in TBS with 0.25% Triton-X (TBS-T). An ABC Elite kit (Vector Laboratories Inc., Burlingame, CA) was used to visualize immunopositivity signals. Images were obtained using a microscope (Carle Zeiss). Aβ plaques were evaluated as the percentage of the immunostained area (positive pixels) with respect to the total area examined (total pixels) using ImageJ (NIH, Bethesda, MD, USA).

### Western blot analysis

Cortical tissues were homogenized in lysis buffer containing a cocktail of phosphatase and protease inhibitors. A BCA protein assay kit (Pierce, Rockford, IL, USA) was used to determine the protein concentrations for each sample. The same amounts of protein were subjected to SDS polyacrylamide gel electrophoresis (SDS-PAGE) and separated proteins were immunoblotted on polyvinylidene difluoride (PVDF) membranes (Millipore), followed by incubation with primary antibodies at 4 °C overnight after blocking with 5% skim milk. The membranes were washed and incubated with an appropriate horseradish peroxidase-conjugated secondary antibody. Immuno Star Zeta or Immuno Star LD (Wako) was used to visualize immunoreactive bands, which were analyzed with an Amersham Imager 680 (GE Healthcare Life Science). Signal intensity was quantified using the ImageJ (NIH) program (Bethesda, MD, US). The primary and secondary antibodies used are shown in Supplementary Table 1.

### Statistical analyses

Statistical analyses were carried out using GraphPad Prism software (GraphPad Software, San Diego, CA). Data are presented as the mean ± SD. Significant differences among groups were assessed by Student’s t-test, analysis of variance (ANOVA), and repeated measures ANOVA with Fisher’s PLSD test.

### Ethics for animal experiments

This study was performed in strict accordance with the ARRIVE guidelines (Animal Research: Reporting in vivo Experiments) for the Care and Use of Laboratory Animals of the National Institutes of Health. All procedures were performed according to the guidelines and regulations and approved by the Committees on Nagoya City University Institutional Care and Use of Laboratory Animals (Approval Number: H29M-022H05) and the Animal Experimentation Committee of Chubu University (Approval Numbers: #1910037, #2010014, #2110009, #2210037, #2310028).

## Supplementary Information

Supplementary Information.
