# Supplementary material for: Sustained high body temperature exacerbates cognitive function and Alzheimer’s disease-related pathologies
Source: Sci Rep. 2022 Jul 18;12:12273. doi: 10.1038/s41598-022-16626-0 (PMC9293958; doi:10.1038/s41598-022-16626-0)
Supplement: Supplementary file 1 — Supplementary Information. [file 41598_2022_16626_MOESM1_ESM.pdf]

Sustained high body temperature exacerbates cognitive function and Alzheimer's disease-related pathologies

Cha-Gyun Jung[1, §], Reiko Kato[2], Chunyu Zhou[1], Mona Abdelhamid[1], Esraa Ibrahim A. Shaaban[1], Hitoshi Yamashita[2, §], Makoto Michikawa[1, §].

[1] Department of Biochemistry, Graduate School of Medical Sciences, Nagoya City University, Nagoya, Japan
[2] Department of Biomedical Sciences, College of Life and Health Sciences, Chubu University, Kasugai, Japan.

Supplementary Table 1. List of antibodies used in the study.

Antibodies	Species	Dilution	Source	Cat. No.
Primary antibodies				
ABCA1	Mouse	1:1000	Abcam	AB18180
ADAM10	Rabbit	1:1000	Millipore	AB19026
ApoE	Goat	1:1000	Millipore	AB947
APP	Mouse	1:1000	Millipore	MAB348
BACE1	Mouse	1:1000	R&D	MAB931
APP-CTF	Rabbit	1:1000	Sigma	A8717
IDE	Rabbit	1:1000	Covance	PRB-282C
NEP	Goat	1:1000	R&D	AF1126
PS1	Mouse	1:1000	Millipore	MAB5232
sAPPβ	mouse	1:1000	IBL	10321
total tau	Mouse	1:1000	BioLegend	806401
Tau p-S199	Rabbit	1:1000	Invitrogen	44734ZG
Tau p-T212/S214	Mouse	1:1000	Innogenetics	90209
Tau p-S404	Rabbit	1:1000	Invitrogen	44758ZG
Tau p-S422	Rabbit	1:1000	Invitrogen	44764ZG
HSP27	Rabbit	1:1000	Bioworld	BS3435
HSP60	Mouse	1:2000	BD Biosciences	611562
HSP70	Rabbit	1:2000	Bioworld	BS2741
HSP 90	Mouse	1:2000	BD Biosciences	610418
Total GSK3β	Rabbit	1:1000	Cell Signaling	9332
p-GSK3α/β (T279/Y216)	Rabbit	1:2000	Invitrogen	44604G
Total SAPK/JNK	Rabbit	1:1000	Cell Signaling	9252
p-SAPK/JNK T183/Y185	Rabbit	1:1000	Cell Signaling	9251
Total ERK	Rabbit	1:2000	Cell Signaling	9102
p-ERK T202/Y204	Rabbit	1:2000	Cell Signaling	9101
Total p38	Rabbit	1:1000	Cell Signaling	9211
p-p38 T180/Y182	Rabbit	1:1000	Cell Signaling	9212
α-Tubulin	Rabbit	1:2000	Cell Signaling	2148
Secondary antibodies				
Anti-rabbit IgG, HRP-linked	Goat	1:5000	Cell Signaling	7074
Anti-mouse IgG, HRP-linked	Mouse	1:5000	Cell Signaling	7076

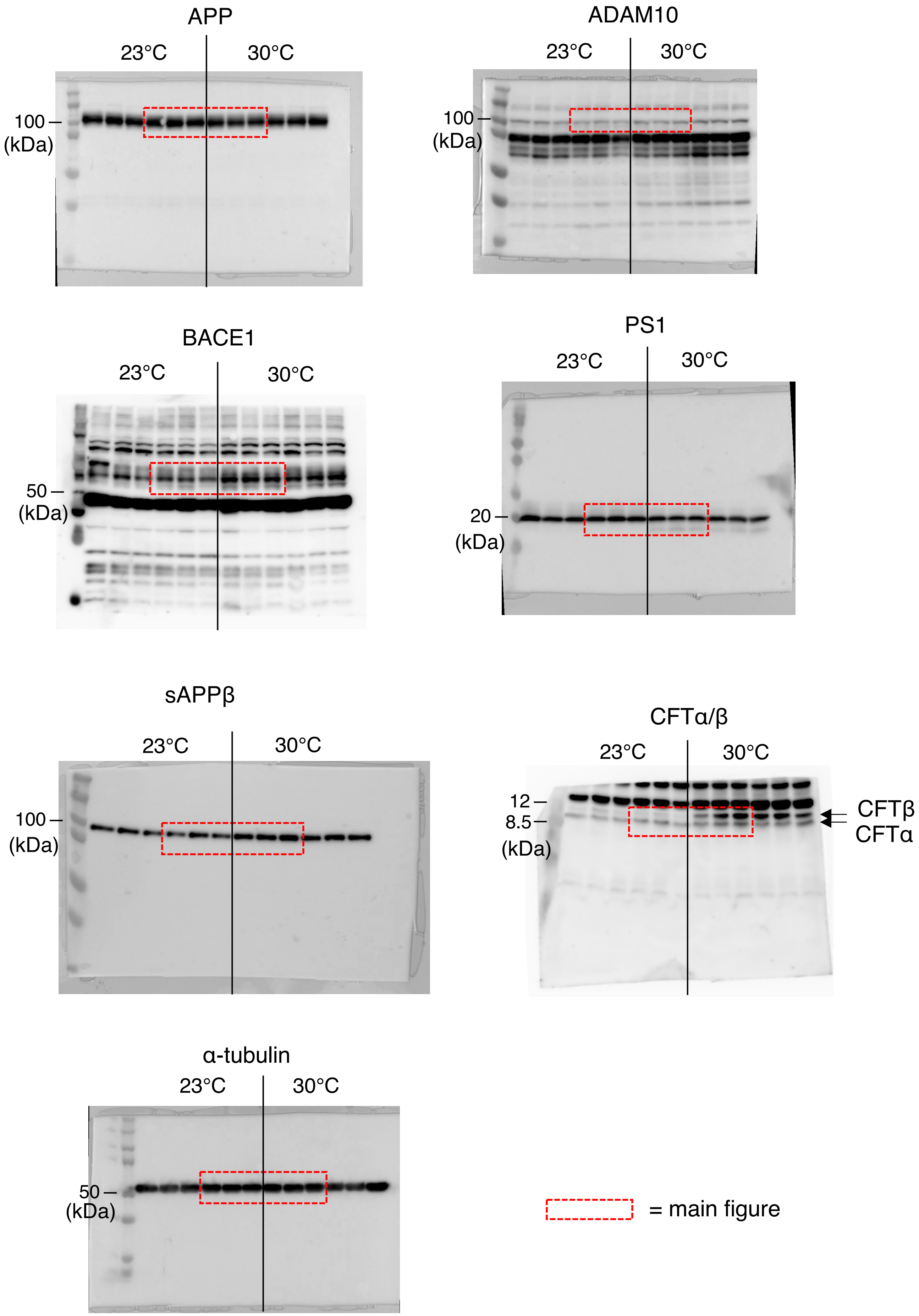
APP
23°C
30°C
100
(kDa)
ADAM10
23°C
30°C
100
(kDa)
BACE1
23°C
30°C
50
(kDa)
PS1
23°C
30°C
20
(kDa)
sAPPβ
23°C
30°C
100
(kDa)
CFTα/β
23°C
30°C
12
8.5
(kDa)
CFTβ
CFTα
α-tubulin
23°C
30°C
50
(kDa)
= main figure

Fig. 3

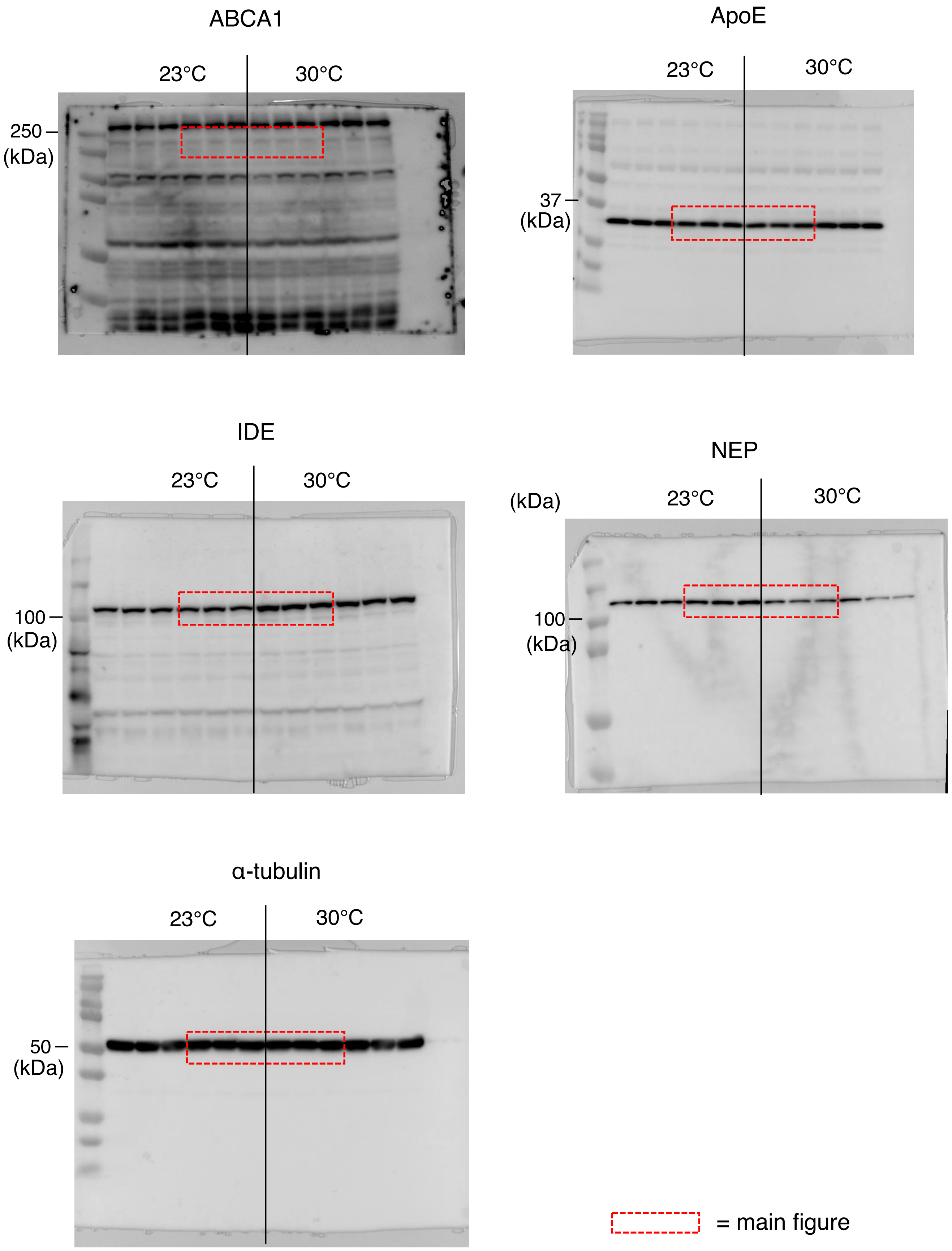
ABCA1
23°C
30°C
250
(kDa)
ApoE
23°C
30°C
37
(kDa)
IDE
23°C
30°C
100
(kDa)
NEP
(kDa)
23°C
30°C
100
(kDa)
α-tubulin
23°C
30°C
50
(kDa)
= main figure

Fig. 4

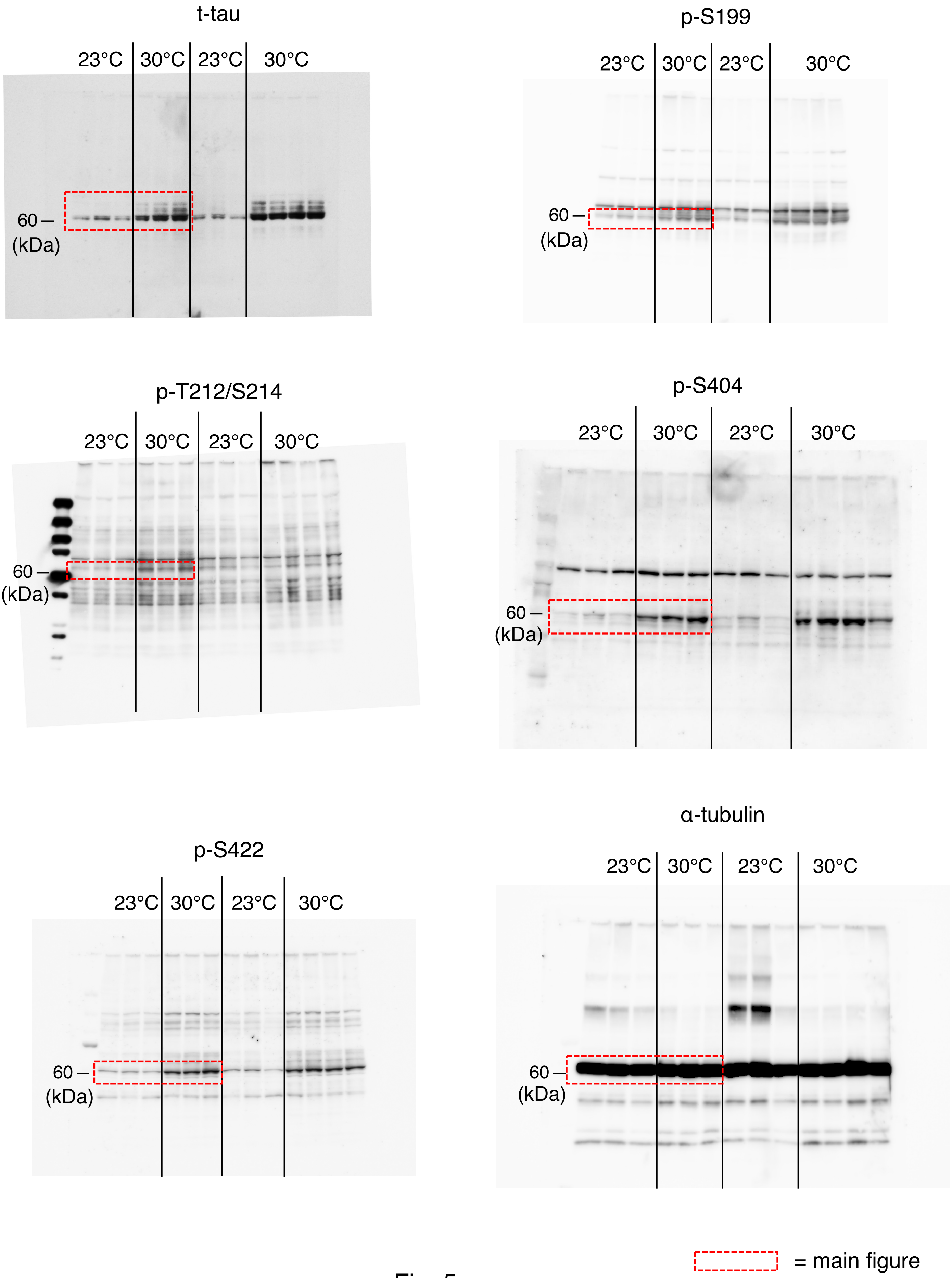
t-tau
23°C 30°C 23°C 30°C
60 —
(kDa)
p-S199
23°C 30°C 23°C 30°C
60 —
(kDa)
p-T212/S214
23°C 30°C 23°C 30°C
60 —
(kDa)
p-S404
23°C 30°C 23°C 30°C
60 —
(kDa)
p-S422
23°C 30°C 23°C 30°C
60 —
(kDa)
α-tubulin
23°C 30°C 23°C 30°C
60 —
(kDa)
= main figure

Fig. 5a

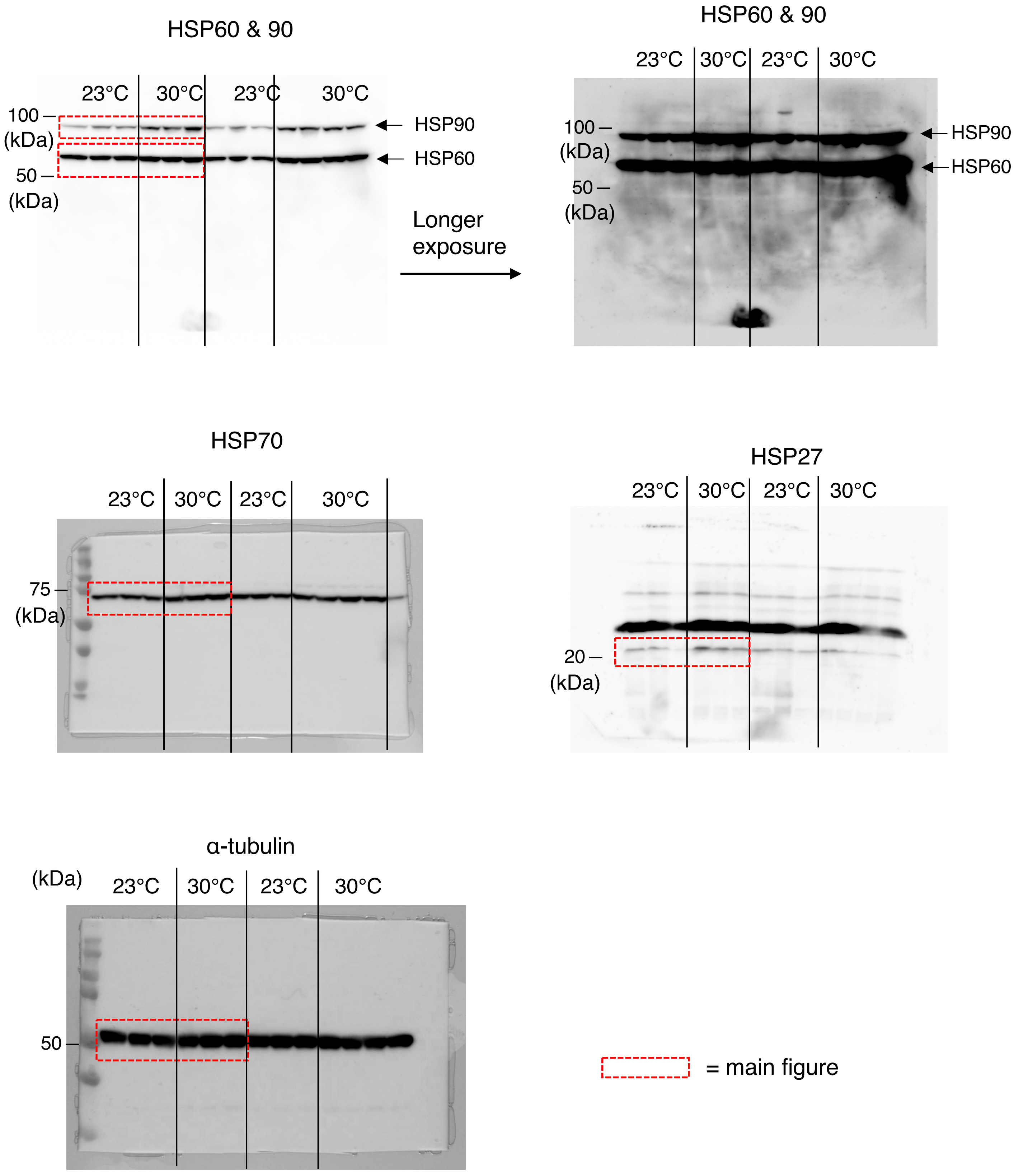

HSP60 & 90
23°C
30°C
100 —
(kDa)
50 —
(kDa)
HSP90
HSP60
Longer exposure
HSP70
75 —
HSP27
20 —
α-tubulin
50 —
= main figure

Fig. 5b

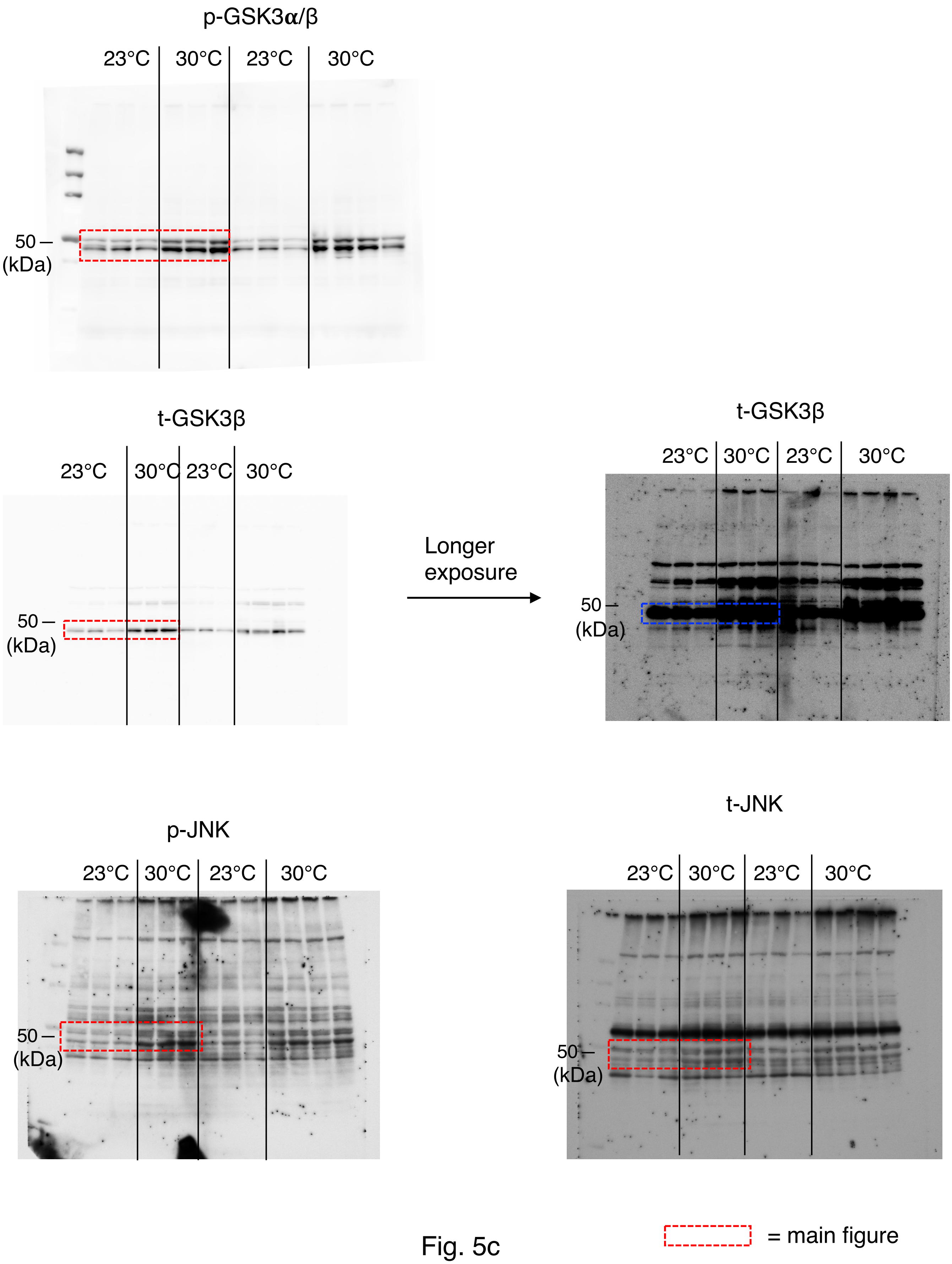
p-GSK3α/β
23°C
30°C
23°C
30°C
50 —
(kDa)
t-GSK3β
23°C
30°C
23°C
30°C
50 —
(kDa)
Longer exposure
t-GSK3β
23°C
30°C
23°C
30°C
50 —
(kDa)
p-JNK
23°C
30°C
23°C
30°C
50 —
(kDa)
t-JNK
23°C
30°C
23°C
30°C
50 —
(kDa)
= main figure

Fig. 5c

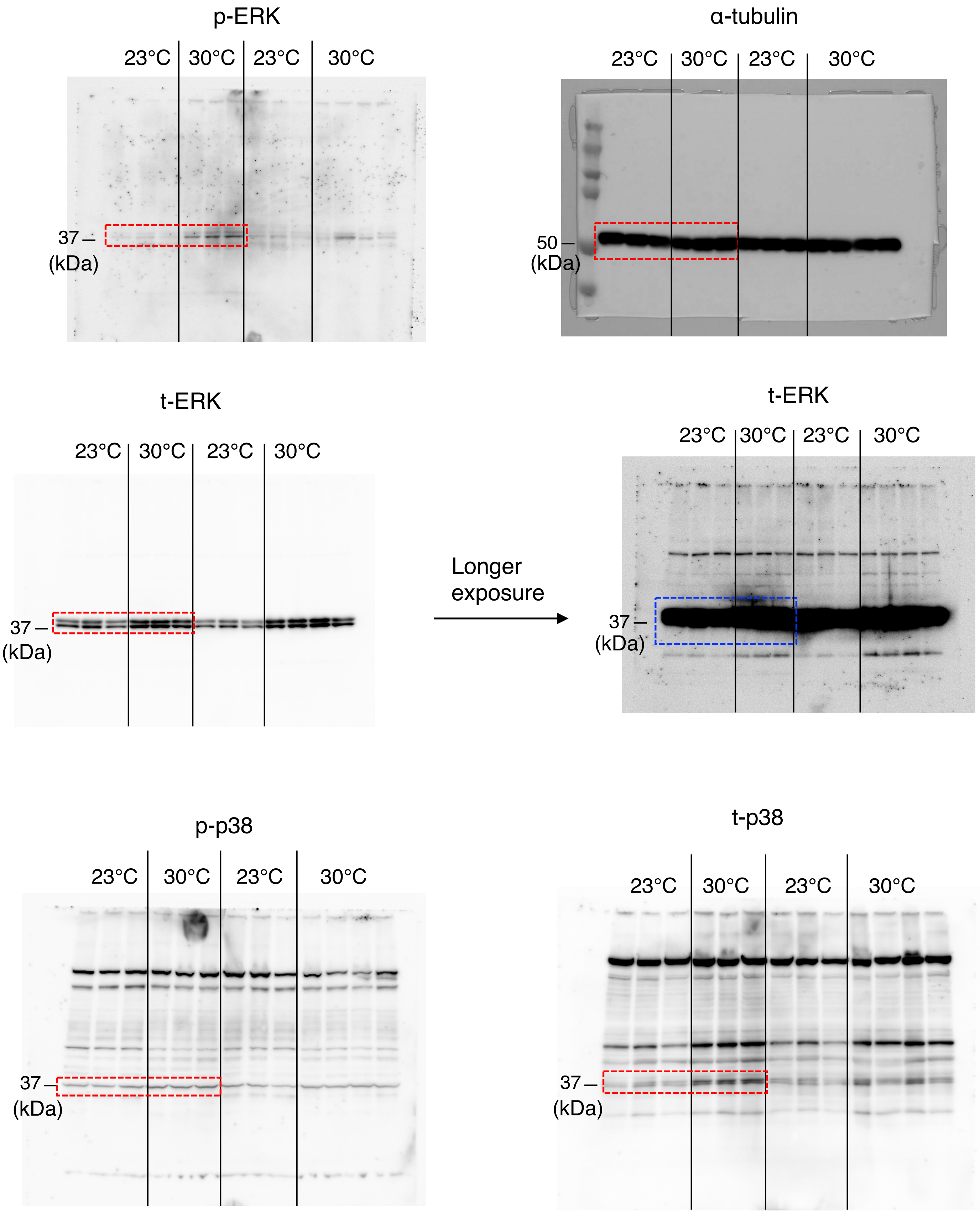

Fig. 5c

= main figure
